# Multiorgan Failure Secondary to Intentional Acetaminophen Overdose-Induced Methemoglobinemia

**DOI:** 10.7759/cureus.83833

**Published:** 2025-05-10

**Authors:** Mohamed A Abutineh, Chirag Lodha, George Mitchell

**Affiliations:** 1 Internal Medicine, Edward Via College of Osteopathic Medicine, Spartanburg, USA; 2 Critical Care Medicine, Cleveland Clinic Indian River Hospital, Vero Beach, USA

**Keywords:** acetaminophen overdose, drug overdose, medical icu, methemoglobinemia, multiorgan system failure, toxin induced methemoglobinemia

## Abstract

Although acetaminophen toxicity has been reported to cause methemoglobinemia, its recognition remains limited in the clinical literature. Methemoglobinemia often necessitates a high index of clinical suspicion, as it may contribute to lactic acidosis and multiorgan dysfunction due to impaired tissue oxygenation. A 21-year-old man presented to the emergency department (ED) via emergency medical services (EMS) with reports of an intentional overdose of an unknown amount of bupropion, two pill bottles of acetaminophen, and an unknown amount of bleach. The patient was emergently intubated. Despite reported bleach ingestion, esophagogastroduodenoscopy (EGD) revealed no evidence of caustic injury or esophagitis. The poison center was contacted, and the patient was started on N-acetylcysteine (NAC). The exact time of acetaminophen ingestion was unknown; however, liver function tests were normal at presentation. Transaminases became abnormal 48 hours later (well after NAC administration had begun). Persistent lactic acidosis in the context of normal initial transaminase levels raised clinical suspicion for methemoglobinemia, given the potential for tissue hypoxia. Methemoglobin levels were confirmed to be elevated, potentially explaining tissue ischemia. The patient received methylene blue as the antidote. The liver transplant team was consulted and agreed with the poison center's recommendation of excluding acetaminophen-induced liver injury. Due to unexplained elevated lactic acid and multisystem organ failure, the family elected for a Do Not Resuscitate (DNR) status. The patient expired four days later with multisystem organ failure.

## Introduction

Acetaminophen toxicity, pathogenesis, and treatment

Acetaminophen, also known as paracetamol in Europe, is a commonly used over-the-counter analgesic and antipyretic due to its favorable safety profile at therapeutic doses and low cost. Because of its widespread availability, acetaminophen is the leading cause of both unintentional and intentional drug overdoses in the United States [1]. It is also the most common cause of acute liver failure (ALF) in many countries worldwide [2]. In overdose patients, mortality rates have been approximated at 0.4%, or about 300 deaths annually in the United States alone [3].

The mechanisms underlying acetaminophen-induced hepatotoxicity are well characterized in the literature. The majority (about 90%) of acetaminophen undergoes conjugation by uridine diphosphate (UDP)-glucuronosyltransferases (UGT) and sulfotransferase (SULT), forming glucuronidated and sulfated metabolites, which are excreted in the urine [4]. A smaller amount (about 10%) of acetaminophen is oxidized by the hepatic cytochrome CYP 2E1 to N-acetyl-para-benzo-quinone imine (NAPQI), a highly toxic metabolite [5]. NAPQI is subsequently conjugated by glutathione (GSH) into cysteine conjugates and mercapturic acid (non-toxic). During acetaminophen overdose, excessive NAPQI formation surpasses the liver’s GSH detoxification capacity, resulting in oxidative stress, mitochondrial injury, and subsequent hepatocellular necrosis [6]. Acetaminophen toxicity is known to be dose-dependent; at toxic doses, the glucuronidation and sulfation pathways become more saturated, and more acetaminophen is metabolized to NAPQI [7]. In some cases, GSH depletion also impairs the γ-glutamyl cycle, promoting the accumulation of 5-oxoproline (pyroglutamic acid), which has been associated with high anion gap metabolic acidosis.

The clinical presentation of patients with acetaminophen overdose can be varied depending on the formulation (extended-release, opiate-acetaminophen combinations, etc.), dose, co-ingestants (alcohol, supplements), and pre-existing liver disease. Generally, there are four established sequential stages of acetaminophen hepatotoxicity [8]. Stage I occurs within the first 24 hours of ingestion and is characterized by nonspecific symptoms resembling a viral prodrome (malaise, nausea, vomiting), with aspartate aminotransferase (AST) and alanine transaminase (ALT) usually at normal levels. Stage II occurs within 1-3 days and is characterized by the elevation of AST and ALT, jaundice, and tender hepatomegaly. Stage III occurs within 3-4 days and is the point at which liver injury is maximal, with extensive AST and ALT elevations, jaundice, encephalopathy, coagulopathy, and lactic acidosis. The highest risk of mortality is at this stage and frequently occurs due to multiorgan failure, with lactic acidosis being a poor prognostic factor. The mechanism of lactic acidosis is elucidated to be due to tissue hypoxia and decreased hepatic metabolism of lactate. Also, 5-oxoproline (pyroglutamic acid) has been reported in the literature as a cause of anion gap metabolic acidosis and subsequent renal failure after acute acetaminophen overdose [9-10]. Stage IV occurs after 96 hours and may last 1-2 weeks and is marked by recovery.

N-acetylcysteine (NAC) is considered the antidote for acetaminophen hepatotoxicity, which is protective if given within the first eight hours after ingestion [11]. NAC provides cysteine and replenishes intracellular GSH levels in the liver, allowing for the detoxification of NAPQI. Determining factors of acetaminophen toxicity severity are two-fold: the ingested dose and the length of time from acetaminophen ingestion to N-acetylcysteine therapy (“time-to-NAC") [12]. If clinical deterioration continues, liver transplantation is the definitive therapy.

Methemoglobinemia: pathogenesis and treatment

Every molecule of hemoglobin contains four iron atoms, with each iron atom having an atomic valence of +2 (referred to as ferrous ion). If hemoglobin is exposed to an agent that oxidizes it (removing an electron), the iron atom adopts a +3 atomic valence, then referred to as a ferric ion. Hemoglobin, which has one or more of its iron atoms in the ferric state, is referred to as methemoglobin. Ferric hemes cannot bind oxygen. Instead, the remaining ferrous hemes in the hemoglobin molecule have increased affinity for their bound oxygen and therefore do not readily release it to peripheral tissues (resulting in a leftward shift of the oxyhemoglobin dissociation curve) [13].

Methemoglobinemia can be acquired from exogenous substances. Commonly implicated medications include topical anesthetic agents such as benzocaine, lidocaine, and prilocaine [14-15]. Topical anesthetics, commonly used for various diagnostic procedures, are well-documented inducers of methemoglobinemia. In a retrospective series of 138 cases of methemoglobinemia, dapsone was the implicated drug 42% of the time [16]. Antimalarial drugs such as chloroquine [17], primaquine, and more recently hydroxychloroquine [18] have been associated with methemoglobinemia. Nitrates and nitrites from foods, drugs, preservatives, and well water have also been linked to acquired methemoglobinemia [19].

Cyanosis with normal oxygen saturation and absence of pathology in the respiratory or cardiovascular systems should raise clinical suspicion for methemoglobinemia. Nonspecific symptoms such as headache, lightheadedness, and fatigue are often present, but shock, severe respiratory depression, and clinical deterioration due to tissue hypoxia can also be present [20]. Methemoglobin is detected in blood gas from either arterial or venous samples and is expressed as a percentage. Methylene blue is the standard of care for the treatment of elevated methemoglobin levels [20]. It acts by accepting an electron from nicotinamide adenine dinucleotide phosphate hydrogen (NADPH), turning into leukomethylene blue, and then reducing the 3+ ferric state back to the 2+ ferrous state in red blood cells. Methylene blue should be avoided in patients with glucose-6-phosphate dehydrogenase (G6PD) deficiency, as it may worsen methemoglobinemia and precipitate hemolytic anemia.

Acetaminophen-induced methemoglobinemia

Methemoglobinemia with acetaminophen toxicity has been reported in exceedingly rare cases, but a mechanism is yet to be clearly defined [21]. A systematic review found that between 1968 and 2019, there were only 14 reported cases of acetaminophen-induced methemoglobinemia in the literature [22]. Acquired methemoglobinemia from acute poisoning of any drug due to intentional self-harm can be difficult to recognize because the condition is rare: among a group of 828 individuals hospitalized for different drug intoxications due to intentional self-harm, only seven patients (0.84%) were found to have methemoglobinemia [23].

## Case presentation

A 21-year-old African American male presented to the emergency department (ED) via emergency medical services (EMS) and was responsive to painful stimuli only. The patient vomited copious amounts of light pink, frothy material, with oval-shaped light blue capsules identified in the emesis. The patient had been found unresponsive at home by his guardian, who performed cardiopulmonary resuscitation (CPR). It is unclear if he was in cardiac arrest. The patient’s guardian had called and told ED staff that the patient ingested two bottles of acetaminophen, an unknown amount of bupropion, and an unknown amount of bleach. The patient arrived in the ED with an unlabeled medication bottle. A toxicology screening was performed, which ruled out other co-ingestants.

Upon admission, the patient's vital signs were as follows: blood pressure 112/51 mmHg, pulse 104 beats per minute, temperature 93.6 °F, respiratory rate 22 breaths per minute, and oxygen saturation 95%. He received etomidate and rocuronium for intubation and was started on propofol and fentanyl infusion titrated to standard doses in conjunction with the critical care pharmacy team. Levetiracetam was initiated for seizure prophylaxis. The poison center was contacted. The patient was monitored for seizure activity via continuous electroencephalogram (EEG). Computed tomography (CT) of the abdomen/pelvis and chest with intravenous (IV) contrast imaging was negative for evidence of esophageal perforation or esophageal mural thickening. The patient had one generalized tonic-clonic seizure event during the CT which was alleviated by lorazepam 2 mg. Head CT without IV contrast was negative for evidence of intracranial abnormalities. An electrocardiogram (ECG) was significant for sinus tachycardia and a prolonged QT interval of 544 msec. The patient was seen in consultation by neurology, nephrology, and toxicology during hospitalization. NAC therapy was initiated per poison center recommendations beginning with a loading dose of 15,560 mg in D5W 1000 mL (200 mg/kg/dose). Esophagogastroduodenoscopy (EGD) was negative for evidence of damage due to the reported ingestion of bleach. A dialysis catheter was inserted 24 hours after hospitalization; however, the patient only received one session of hemodialysis. A right upper quadrant abdominal ultrasound showed no acute abnormalities. The patient underwent hemodialysis to correct worsening acidosis, which did improve, but liver function tests (LFTs) worsened despite the initiation of the NAC protocol. This prompted an investigation of methemoglobin levels, which were elevated at 4.5% (reference range: <1.2%). He then received methylene blue 100 mg in D5W 50 mL for methemoglobinemia, which subsequently improved. A chest X-ray, performed 48 hours after hospitalization, was significant for findings suggestive of congestive heart failure, moderate basilar predominant infiltrates likely representing pulmonary edema, and a small to moderately sized right pleural effusion. Previous chest X-rays available for comparison had been clinically insignificant. The patient’s clinical status continued to deteriorate into multiorgan failure. A timeline of the associated laboratory values can be found in Table 1. The intensivist discussed the case with Cleveland Clinic Weston hepatology; the patient was not a candidate for an acute liver transplant, especially given his psychiatric history and unknown neurological status at that point. Further discussions with the patient's guardian led to Do-Not-Resuscitate (DNR) status and Our Legacy organ transplant was notified and reached out to the guardian per guidelines. The patient's guardian was agreeable to organ donation. The patient was extubated and transferred to the operating room (OR) for organ procurement.

**Table 1:** Laboratory values at admission, 48 hours after admission, and before expiration BUN: blood urea nitrogen; Alk Phos: alkaline phosphatase; ALT: alanine transaminase; AST: aspartate aminotransferase; eGFR: estimated glomerular filtration rate; WBC: white blood cells; RBC: red blood cells; Hb: hemoglobin; Hct: hematocrit

Labs	October 4, 2023-October 5, 2023 (admission)	October 7, 2023 (48 hours later)	October 8, 2023 (death)	Reference ranges
Sodium	141 mEq/L	131 mEq/L	132 mEq/L	136-145 mEq/L
Potassium	3.1 mEq/L	5.9 mEq/L	4.2 mEq/L	3.5-5.0 mEq/L
Chloride	104 mEq/L	90 mEq/L	85 mEq/L	98-106 mEq/L
CO_2_	13 mEq/L	26 mEq/L	31 mEq/L	23-28 mEq/L
BUN	12 mg/dL	7 mg/dL	11 mg/dL	8-20 mg/dL
Creatinine	0.55 mg/dL	2.29 mg/dL	3.75 mg/dL	0.70-1.30 mg/dL
Glucose	236 mg/dL	132 mg/dL	170 mg/dL	70-99 mg/dL
Protein	7.0 g/dL	5.0 g/dL	3.8 g/dL	5.5-9.0 g/dL
Calcium	8.2 mg/dL	6.3 mg/dL	6.0 mg/dL	8.6-10.2 mg/dL
Phosphorus	1.9 mg/dL	3.3 mg/dL		3.0-4.5 mg/dL
Albumin	4.3 g/dL	3.0 g/dL	2.4 g/dL	3.5-5.5 g/dL
Alk Phos	71 U/L	88 U/L	84 U/L	30-120 U/L
ALT	17 U/L	5,037 U/L	4,434 U/L	10-40 U/L
AST	24 U/L	5,983 U/L	5,432 U/L	10-40 U/L
Anion gap	24 mEq/L	15 mEq/L	16 mEq/L	7-13 mEq/L
Amylase			332 U/L	25-125 U/L
Lipase			221 U/L	10-140 U/L
eGFR	145 mL/min/1.73 m^2^	41 mL/min/1.73 m^2^	22 mL/min/1.73 m^2^	>60 mL/min/1.73 m^2^
Ammonia	<11 μg/dL			40-70 μg/dL
Lactate	9.9 mmol/L	7.8 mmol/L	7.8 mmol/L	0.7-2.1 mmol/L
Acetaminophen	307 mcg/mL	72 mcg/mL	72 mcg/mL	<20 mcg/mL
Ethanol	<11 mg/dL			<0.005% (<5 mg/dL)
WBC	13.00 x 10^9^/L	1.29 x 10^9^/L	2.12 x 10^9^/L	4-11 x 10^9^/L
RBC	4.48 x 10^12^/L	4.03 x 10^12^/L	2.78 x 10^12^/L	4.2-5.9 x 10^12^/L
Hb	13.3 g/dL	11.9 g/dL	8.4 g/dL	14-18 g/dL
Hct	38.1%	33.3%	22.6%	42%-50%
Platelets	186 x 10^9^/L	83 x 10^9^/L	49 x 10^9^/L	150-450 x 10^9^/L
pH	7.2	7.32	7.49	7.38-7.44
pCO_2_	33 mm Hg	56 mm Hg	47 mm Hg	38-42 mm Hg
pO_2_	262 mm Hg	51 mm Hg	126 mm Hg	75-100 mm Hg
Methemoglobin	2.7%	4.5%	1.4%	<1.2%

The patient’s past medical history included multiple hospitalizations for suicidal ideation in 2021, 2022, and 2023. He had previous mixed psychiatric diagnoses, including bipolar disorder, impulse control disorder, and paranoid schizophrenia. His history is also significant for episodes of self-mutilation. Medications before the current admission included olanzapine 5 mg tablet by mouth every day (PO Qdaily) and fluoxetine 20 mg PO Qdaily, with trials of alternative psychiatric medications having been initiated and discontinued previously. There was no significant surgical history.

The patient’s social history included active tobacco smoking and vaping, infrequent alcohol intake, and active marijuana use. No other illicit drug use per prior history. He was adopted and therefore no family history was able to be elicited. The patient grew up in foster care, and as a young adult met a person who served as his guardian during the course of hospitalization. The patient’s depression had been controlled prior to the current presentation.

## Discussion

The primary concern addressed in this case report is the development of multiorgan failure secondary to methemoglobinemia following acute acetaminophen poisoning. No immediate evidence suggested the involvement of other agents capable of inducing methemoglobinemia. Regarding the patient's bupropion ingestion, methemoglobinemia was not a primary concern. Most of the side effects of bupropion include anxiety, agitation, and possible seizures [24]. The authors attest to excluding bupropion as a mechanism leading to the development of methemoglobinemia. Although this phenomenon has been previously described in case reports, it remains exceedingly rare, particularly in patients without a history of G6PD deficiency. Rianprakaisang et al. previously reported two cases of methemoglobinemia after excessive acetaminophen ingestion in patients with no predisposing disease [21]. Interestingly, Queirós et al. reported a case of hemodialysis-related functional G6PD deficiency that led to methemoglobinemia in a 78-year-old female while taking acetaminophen 3 g/day for seven days [25]. It is possible that our patient experienced a functional G6PD deficiency. Still, patients with G6PD deficiency who are administered methylene blue are known to experience hemolysis [26]. The applicability of this to patients with functional G6PD deficiency has not yet been definitively established in the literature.

The mechanism by which toxic acetaminophen ingestion causes methemoglobinemia in patients without underlying G6PD deficiency is also not well established. Erythrocytes undergo significant oxidative stress due to NAPQI following the GSH-deficient state induced by acetaminophen poisoning. This may be the simplest explanation for methemoglobinemia. A study on acetaminophen-induced methemoglobinemia in cats and dogs proposed that para-aminophenol (PAP), a structural analog of the methemoglobin inducer aniline, is responsible for methemoglobinemia due to a deficiency of arylamine N-acetyltransferase (NAT) activity [27]. However, human erythrocytes contain N-acetyltransferase, responsible for converting PAP to acetaminophen. It may be the case that PAP generation is one factor that lends itself to the increased oxidant stress on erythrocytes.

A second issue highlighted in this case report is the progression of clinical deterioration despite adequate NAC and methylene blue therapy. As previously mentioned, timely NAC administration normally results in virtually complete protection against liver damage [11]. An obvious limitation of NAC and methylene blue therapy is time-to-administration. It is not known how long before presentation the patient ingested toxic amounts of the drug. However, the authors propose that acetaminophen-induced liver injury is less likely to have occurred due to the patient’s normal liver enzymes on presentation and delayed elevation until 48 hours later. In this case, it is more likely that methemoglobinemia decreased oxygen delivery to multiple organs, including the liver, leading to lactic acidosis.

Early treatment with NAC in patients with toxic ingestion of acetaminophen is known to prevent renal impairment and death [11]. Our patient developed kidney failure during the hospitalization, which could be due to multiple mechanisms. In the setting of GSH depletion from acetaminophen toxicity, pyroglutamic acid can accumulate and contribute not only to high anion gap metabolic acidosis but also to renal dysfunction due to impaired clearance and increased acid load. Rapid acute kidney injury after hepatic ischemia due to reperfusion injury has previously been described in mice studies [28], presenting a possible explanation. One note of acknowledgment is the development of thrombocytopenia during the patient’s hospitalization. Stravitz et al. previously described the association of thrombocytopenia with multiorgan system failure in patients with ALF [29].

To date, it remains the case that there are no extensive literature reviews that can characterize acetaminophen-induced methemoglobinemia according to dose ingested/serum acetaminophen levels. There is also a clear lack of literature regarding the response to methylene blue in patients with acetaminophen-induced methemoglobinemia. Interestingly, methylene blue administration has also been associated with unusual greenish-bluish organ discoloration in fatal aluminum phosphide poisoning cases where methylene blue was administered, though the exact biochemical mechanism remains unclear [30]. This phenomenon underscores the need for further research into methylene blue's interactions with toxic agents and its potential tissue-level effects beyond methemoglobin reduction. Clinicians must keep a high index of suspicion for methemoglobinemia due to self-harm-related ingestion of acetaminophen, as it can present as lactic acidosis and multiorgan failure in certain patients.

## Conclusions

Acetaminophen toxicity likely resulted in methemoglobinemia in a patient without known underlying G6PD deficiency. The patient’s clinical course deteriorated into multiorgan failure and lactic acidosis despite timely NAC and methylene blue therapy. Our case emphasizes the importance of recognizing methemoglobinemia as a rare but serious complication of acetaminophen overdose, particularly when presenting with unexplained lactic acidosis or multiorgan dysfunction despite otherwise appropriate early management.

Clinicians must maintain a high index of suspicion, especially when the timeline of ingestion is unclear and traditional markers of acetaminophen-induced hepatotoxicity are delayed or absent. Greater awareness and investigation into this underrecognized complication may help inform future therapeutic strategies and improve prognostication in similar cases.
